# Putative Invertebrate, Plant, and Wastewater Derived ssRNA Viruses in Plankton of the Anthropogenically Impacted Anacostia River, District of Columbia, USA

**DOI:** 10.1264/jsme2.ME21070

**Published:** 2022-03-10

**Authors:** Caroline Solomon, Ian Hewson

**Affiliations:** 1 School of Science, Technology, Accessibility, Mathematics and Public Health, Gallaudet University, 800 Florida Ave NE, Washington, DC 20002 USA; 2 Department of Microbiology, Cornell University, Wing Hall 403, Ithaca NY 14853 USA

**Keywords:** RNA, virome, Picornavirales, Tobamovirus, Anacostia, virioplankton

## Abstract

The Anacostia River is a highly impacted watershed in the Northeastern United States which experiences combined sewage outfall in downstream waters. We examined the composition of RNA viruses at three sites in the river using viral metagenomics. Viromes had well represented Picornaviruses, Tombusviruses, Wolframviruses, Nodaviruses, with fewer Tobamoviruses, Sobemoviruses, and Densoviruses (ssDNA). Phylogenetic ana­lyses of detected viruses provide evidence for putatively autochthonous and allochthonous invertebrate, plant, and vertebrate host origin. The number of viral genomes matching Ribovaria increased downstream, and assemblages were most disparate between distant sites, suggesting impacts of the combined sewage overflows at these sites. Additionally, we recovered a densovirus genome fragment which was highly similar to the Clinch ambidensovirus 1, which has been attributed to mass mortality of freshwater mussels in Northeastern America. Taken together, these data suggest that RNA viromes of the Anacostia River reflect autochthonous production of virus particles by benthic metazoan and plants, and inputs from terrestrial habitats including sewage.

Viruses play ecologically and biogeochemically significant roles in aquatic habitats (Fuhrman, 1999; Suttle, 2005). Virioplankton diversity has been examined across a wide range of aquatic habitats from rivers to the central gyres by viral metagenomics (Breitbart *et al.*, 2002; Breitbart *et al.*, 2004; Brum *et al.*, 2015). These studies have revealed an extensive viral taxonomy which exceeds putative host diversity. Despite a growing understanding of virioplankton diversity, there remain important questions concerning the origin of viruses and their putative hosts. While the overwhelming majority of virioplankton particles originate from bacterioplankton and less so from protistan plankton (*e.g.* phytoplankton and heterotrophic protists; Lang *et al.*, 2004; Culley *et al.*, 2006; 2014), viruses in plankton may also originate from metazoans (*e.g.* zooplankton and megafauna; Hewson *et al.*, 2014; Chang, T., *et al.* 2021. Arthropods and the evolution of RNA viruses. *bioRxiv* https://doi.org/10.1101/2021.05.30.446314) and be present in environmental DNA (eDNA) (Kaganer *et al.*, 2021). In shallow rivers and lakes, virioplankton may also originate from adjacent watersheds (*i.e.* allochthonous viruses; Hewson *et al.*, 2012; 2018; Green *et al.*, 2018) or potentially from benthic organisms that release particles into overlying waters. Anthropogenically impacted rivers—*e.g.* those receiving sewage and stormwater inputs may also contain human viruses or those that infect domesticated animals (Tamaki *et al.*, 2012; Hata *et al.*, 2014; Williamson *et al.*, 2014; Bibby *et al.*, 2019; Li *et al.*, 2021). There have to date been few studies of viral diversity in anthropogenically impacted urban rivers (Van Rossum *et al.*, 2015; Adriaenssens *et al.*, 2021).

The Anacostia River that flows through Maryland and Washington, D.C., United States is a short (~14‍ ‍km) tidal freshwater tributary river (leading to the larger Potomac River) that receives inputs from a small, predominately urban catchment (283‍ ‍km^–2^) which hosts one of the most densely populated regions of the United States. The river has historically received anthropogenic inputs from both industrial and military sites (Hwang and Foster, 2006; Velinsky *et al.*, 2011), and currently receives both stormwater and at times untreated sewage, especially in the lower portion of the river during storm events (Velinsky *et al.*, 2011). The residence time for water in the river can be more than 100 days during annual dry periods (Solomon *et al.*, 2019), which has resulted in amplification of pollution (Hoch, 1996). Benthic communities in the Anacostia River historically include bivalve freshwater mussels, which are currently under restoration (Harrison, 2019; Anacostia Watershed Society, 2021). Hence, the Anacostia River provides an ideal study site to survey RNA viruses as they relate to sewage inputs and allochthonous viruses.

The aim of this survey was to examine RNA virioplankton along the Anacostia River to provide context for restoration efforts underway in the watershed. Our results demonstrate that downstream sites bear the greatest number and most different RNA viruses compared to upstream sites, and that these assemblages may include viruses of potential invertebrate, plant, and wastewater origin.

## Materials and Methods

**Fig. 1.**
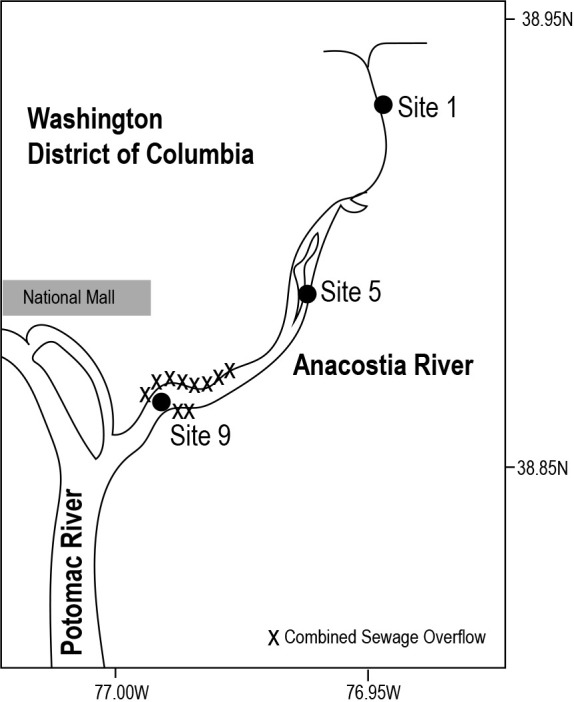
Map of Anacostia River showing location of sampling sites, urban environments, and combined sewage overflows in Washington DC.

Surface water samples were collected at three sites (Table 1) on October 28, 2016 on the Anacostia River (Fig. 1) using a bucket and stored in acid-washed bottles until processing in the lab. Upon arrival at the lab, water samples (between 275–425‍ ‍mL) were serially filtered using a peristaltic pump through 0.2‍ ‍μm Sterivex filters (Millipore) and then 25–30‍ ‍mL through 0.02‍ ‍μm Anotop-25 filters, which were stored at –80°C until ana­lysis at Cornell University. Additional samples for quantification of viral genotypes were collected at each site using the same approach on 27 March 2017, 31 May 2017, 14 July 2017, 27 July 2017 and 28 September 2017.

Viral metagenomes were prepared from the 0.02‍ ‍μm Anotop filters by first extracting DNA and RNA from filters using the Duet DNA/RNA Kit (Zymo Research). Buffer ZR (750‍ ‍μL) was introduced into the sample port of the filter housing, which was capped on the outflow port by a heat-sealed pipette tip. After a 10‍ ‍min incubation to allow for disruption of capsids, the ZR buffer was expunged from the filter housing using positive air pressure from a sterile syringe. All further extraction steps followed the manufacturer’s recommendations. Extracted viral RNA was amplified by the TransPlex whole transcriptome amplification kit 2 kit (WTA2; Sigma-Aldrich). Resulting amplicons were sequenced on Illumina MiSeq 2×150 bp following Nextera library preparation at the Cornell Biotechnology Resource Center (BRC). Three samples were run on a single MiSeq 2×150 bp lane along with 3 virome samples unrelated to this survey (*i.e.* each library comprised ~1/6^th^ of a lane). Sequence data is available at the NCBI under BioProject PRJNA637530; Biosamples SAMN15144738–SAMN15144740; and Short Read Archive SRR11932622–SRR11932625.

Sequence reads were assembled using the CLC Genomics Workbench 10.0 using parameters of 0.5 overlap and 0.8 identity. Bioinformatic ana­lysis followed two workflows. The first workflow compared contiguous sequences (>500 nt) against an in-house database of RNA viral RNA-dependent RNA polymerases (RdRp), coronavirus and flavivirus envelope proteins, and coat proteins of nodaviruses collected from NCBI Genbank on 16 May 2016. Contigs matching these databases by BLASTx (Altschul *et al.*, 1997) at e<10^–10^ were further examined by BLASTn against the non-redundant (nr) database to exclude spurious hits (*i.e.* those which matched cellular microorganism proteins at lower e-value against the nr database). For the second workflow, contig spectra were compared against all Ribovaria proteins deposited in the RefSeq database at NCBI (as of 7 September 2021) by BLASTx, using a cutoff of e<10^–30^. Contigs matching these criteria were then recruited against each read library, and those recruiting >2,000 reads across all 3 libraries proceeded to phylogenetic ana­lysis (see below). Recruitment profiles were scored by presence/absence (due to uncertain biases in the amplification used in this study), where a positive detection comprised >100 reads in any library. The presence/absence matrix for each virus across each library was used to calculate the Bray and Curtis index using XLStat plugin in Microsoft Excel.

To examine the phylogeny of contigs matching Ribovaria as above, read recruitment across the contig was first inspected manually in Geneious Prime to ensure even contig coverage. Next, the open reading frame on each contig was retrieved by the getORF plugin in Geneious Prime, translated into amino acid sequence, and then compared against the nr protein database at NCBI. The 5 closest matches were retrieved for each contig. Because all matches to Ribovaria by this approach were to Picornavirales, a single alignment was performed for all sequences using MUSCLE (Edgar, 2004) with 50 iterations. The alignment was trimmed to remove missing regions on contigs. A phylogenetic reconstruction was performed using maximum likelihood in MEGAX (Kumar *et al.*, 2018). Before phylogenetic reconstruction, the best-fit substation model was determined for each alignment based on lowest Bayesian Information Criterion using all amino acid sites (*i.e.* no gaps), uniform rates across amino acid sites, and with 1,000 bootstrap iterations. The maximum likelihood heuristic model was built by nearest-neighbor interchange, where the initial tree was made by neighbor joining.

Because ssDNA viruses and especially densoviruses are routinely recovered from RNA viromes, likely due to biases in amplification strategy and compatibility of rolling hairpin replication self-complementation (Jackson *et al.*, 2020), we also examined contig spectra for the presence of all Parvoviridae proteins in RefSeq by BLASTx at 1e–30. All matching contigs were then analyzed as described for RNA viruses above.

We also performed phylogenetic reconstruction of two contigs matching Nodaviral and Tobamoviral proteins as described above, and sought to examine their abundance in virioplankton over time and between sites. These two contigs were chosen since they represented the longest contigs matching these families, with greatest coverage, that were recruited reads from all three libraries (*i.e.* the greatest likelihood of detection by quantitative PCR). Quantitative reverse transcriptase PCR (qRT-PCR) primers and TaqMan probes were developed around the two contigs using the web-based program Primer3 (Rozen and Skaletsky, 2000). The sequence of primers, probes and standards are indicated in Supplemental Table 1. The workflow for detection of viral contigs varied from the workflow producing viral metagenomes. Anotop 0.02‍ ‍μm filters were extracted using the Zymo RNA Viral kit (Zymo Research) and eluted in 30‍ ‍μL nuclease-free water including the on-column DNAseI digestion step included in the kit. Extracted RNA was subject to reverse transcription using SuperScript III (Life Biotechnologies Invitrogen) following manufacturer’s protocols including a duplicate for each RNA extract containing no reverse transcriptase (no-RT controls). The reverse transcribed copy DNA (cDNA) and no-RT controls were then used as template in quantitative PCR (qPCR). qPCR reactions contained 1×TaqMan Universal MasterMix (Life Biotechnologies Invitrogen), 200 pmol of each primer and probe and were run in duplicate 25‍ ‍μL reactions with 2‍ ‍μL template cDNA or No RT controls. A third replicate was spiked with 10^4^ copies of the standard to check for qPCR inhibition, and all samples were run against a dilution series from 10^8^ through 10^1^ copies reaction^–1^ of oligonucleotide standard. Each run comprised a 10‍ ‍min heat activation at 94°C, followed by 60 rounds of thermal cycling at 94°C for 15‍ ‍s and 55°C for 1‍ ‍min in a StepOne qPCR machine (Applied Biosystems). Reactions for which both qPCR duplicates provided a quantity above the minimum standards used (*i.e.* >10^3^ copies per reaction) were averaged, and the quantity in the corresponding no RT controls were subtracted from the average value.

## Results and Discussion

Viral metagenomes comprised a total of 16,332,444 paired-end sequence reads, resulting in 14,221 contigs >500‍ ‍nt with the most reads at site 9 (Ana9b) (Table 2). Approximately 21–24% of contigs matched known RNA viruses, which recruited 2–7% of total reads in each library. The dissimilarity (Bray-Curtis Index, BC) was greatest between site 1 (Ana1b) and site 9 (Ana9b; BC=0.53), followed by between site 1 (Ana1b) and site 5 (Ana5b) (BC=0.50), and finally between site 5 and site 9 (BC=0.33) based on positive (here defined as >100 read) recruitment against Ribovaria proteins. These data follow stream-length distance and suggest that sites 5 and 9 experience the greatest input of virioplankton from stormwater and wastewater discharge compared to site 1. This result is also consistent with site 9 having the largest library size overall.

### Taxonomic overview of RNA viral metageomes

Ribovaria realm-affiliated viruses in Anacostia virioplankton by this approach included viruses within the Orthonovirae and Pisuviricota orders Picornavirales (Pisoniviricetes), Solemovirales (Pisoniviricetes), Kitrinoviricota orders Nodamuvirales (Magsaviricetes), Martellivirales (Alsuviricetes), Tolivirales (Tolucaviricetes), and Lenaviricota order Wolframvirales (Ambiliviricetes). We focused our ana­lysis on contigs that were 2,000‍ ‍nt in length with coverage 20× for Picornavirales, and contigs 500 nt in length with 10× coverage for the remaining viral orders. A total of 20 contigs matching Picornavirales, 1 contig matching Solemovirales, 9 contigs matching Nodamuvirales, 1 contig matching Martellivirales, 11 contigs matching Tolivirales, and 9 contigs matching Wolframvirales satisfied these criteria and were subject to phylogenetic ana­lyses. Between sites, most viral genome fragments meeting this criteria were retrieved from site 9 (32 contigs), followed by site 1 (12 contigs) and 5 (11 contigs).

Of contigs matching Picornavirales, 5 were broadly classified within the Marnaviridae, 8 within the Dicistroviridae, while the remainder could not be classified beyond order based on the phylogenetic ana­lysis. Most picornavirus-like contigs matched most closely viral genomes retrieved in transriptome surveys of invertebrates (Shi *et al.*, 2016) or environmental viral metagenomes, however 3 near identical contigs (Dicistrovirus-like Ana1b contig 155, Ana5b contig 75, and Ana9b contig 90), all well recruited from each library, were most similar to viromes retrieved from feces/rectal swabs of birds (Yao *et al.*, 2021) and bats (Bennett *et al.*, 2019) (Supplemental Fig. 1). While the diet of these host sources is not constrained by authors, it is likely that these vertebrates are insectivorous, and hence the dicistroviruses may originate from consumed arthropods. At least one contig (Ana1b contig 306) matched most closely a Picornavirus retrieved from a bivalve transcriptome (Shi *et al.*, 2016). Hence, these data demonstrate that at least some Picornavirales in virioplankton of the Anacostia River likely originate from either terrestrial or aquatic arthropods which inhabit the river.

Our observation of a large number of Picornavirales-like viral genome fragments is consistent with numerous viral metagenomic and metatranscriptomic surveys targeting free virions in freshwater, marine and soil environments (Culley *et al.*, 2006, 2007, 2014; Culley and Steward, 2007; Lang *et al.*, 2009; López-Bueno *et al.*, 2015; Miranda *et al.*, 2016; Allen *et al.*, 2017; Starr *et al.*, 2019; Wolf *et al.*, 2020) (Hillary, L.S., *et al.* 2021. Diverse soil RNA viral communities have the potential to influence grassland ecosystems across multiple trophic levels. *bioRxiv* https://doi.org/10.1101/2021.06.28.448043), as well as tissue-derived viromes of invertebrate (Shi *et al.*, 2016; Hewson *et al.*, 2020), plant (Lachnit *et al.*, 2016) and vertebrate hosts (Li *et al.*, 2010; Bennett *et al.*, 2019; Paskey *et al.*, 2020). The wide detection of picornaviruses in asymptomatic invertebrate specimens suggests that they may rarely cause pathology in their hosts. No picornavirus-like genome fragment retrieved in this survey was most similar to viruses that cause pathology in their hosts.

**Fig. 2.**
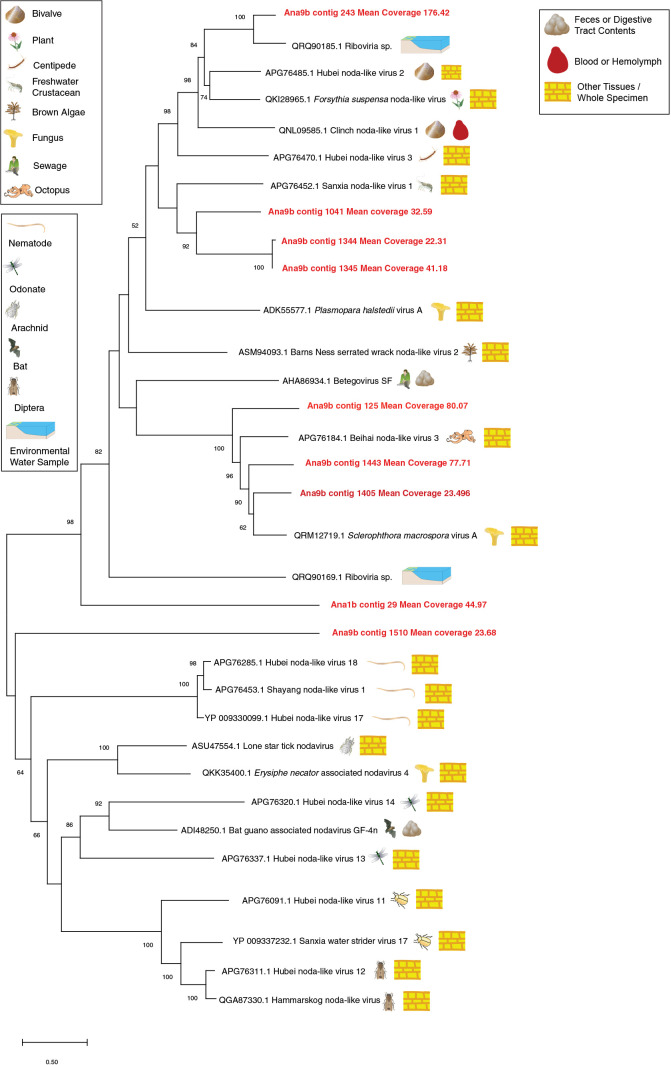
Phylogenetic ana­lyses of Nodamuvirales-like genome fragments (>1,500 nt and at >15× coverage) recovered from the Anacostia River and closest sequences at NCBI. The ana­lysis was based on a 485 amino acid alignment and using the LG model (Le and Gascuel, 2008), a discrete gamma distribution, and assuming a certain fraction of sites are evolutionarily invariable. The putative source/host and sample type of each matching sequence is provided with symbols.

**Fig. 3.**
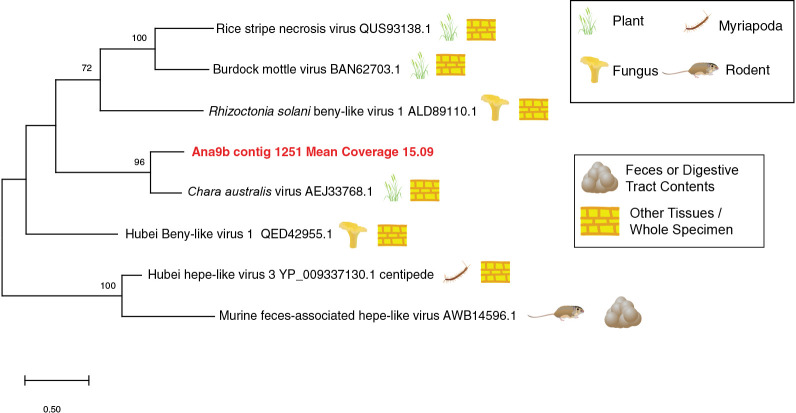
Phylogenetic ana­lyses of Tobamovirus-like genome fragments (>1,500 nt and at >15× coverage) recovered from the Anacostia River and closest sequences at NCBI. The ana­lysis was based on a 103 amino acid alignment and using the WAG model (Whelan and Goldman, 2001) and gamma distribution of rates among sites. The putative source/host and sample type of each matching sequence is provided with symbols.

The sole Solemovirales contig (Ana9b contig 1196) formed a taxonomic cluster with viruses retrieved from mollusks, arachnids, sewage, plants and fungi, and was most similar to luteo-like viruses retrieved from bivalve transcriptomes (Shi *et al.*, 2016) (Supplemental Fig. 2S). All Nodamuvirales were within the Alphanodavirus genus, and were most closely related to viruses retrieved from aquatic arthropods, mollusks and fungi (Fig. 2). The abundance of one Nodamuvirales contig (Ana9b contig 1040) was below detection threshold by qRT-PCR throughout the survey period, suggesting that virome preparation or the sequencing depth was more sensitive to Nodamuvirales. Four tombusvirus (Tolivirales)-like contigs matched most closely viruses infecting plant hosts (Supplemental Fig. 3S). One contig (Ana1b contig 126) matched a tombusvirus recovered from sewage (Greninger and DeRisi, 2015). The remaining tombusviruses recovered matched viruses retrieved from transcriptomes of aquatic and terrestrial invertebrates, including 2 matching most closely bivalve viruses (Shi *et al.*, 2016). The only Martellivirales contig (Ana9b contig 1251) matched most closely *Chara australis* virus (Gibbs *et al.*, 2011) (Fig. 3). Narnaviruses (Wolframvirales) were placed in a broad taxonomy including viruses retrieved from transcriptomes prepared from invertebrates, vertebrates, plants, and environmental soil samples (Supplemental Fig. 4S).

**Fig. 4.**
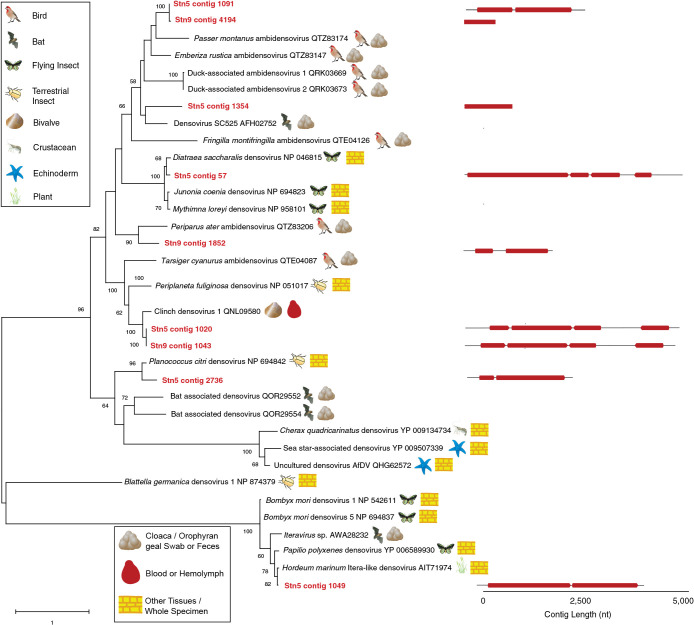
Phylogenetic ana­lyses of Densovirus-like cap-like genome fragments (>500 nt and at >10× coverage) recovered from the Anacostia River and closest sequences at NCBI. The ana­lysis was based on a 176 amino acid alignment and using the WAG model (Whelan and Goldman, 2001) and gamma distribution of rates among sites. The putative source/host and sample type of each matching sequence is provided with symbols. The genome architecture/ORF orientation is given at right.

We also observed several distinct and near-complete genomes of Monodnaviridia (Piccovirales; Quintoviricetes; Cossaviricota; Shotokuvirae) belonging to the Densovirinae, which is consistent with previous observations that RNA viromes prepared using this approach may bear ssDNA genomes (Hewson *et al.*, 2020; Jackson *et al.*, 2020) (Fig. 4). We focused ana­lysis around contigs 500 nt in length with 15× coverage for this order. All Densovirinae genome fragments were most similar to vertebrate fecally-derived (Vibin *et al.*, 2020), insect and molluskan densoviruses (Richard *et al.*, 2020). Of these, most had amino acid similarity and genome architecture consistent with the Ambidensovirus genus, but at least one was most similar to the genus Iteravirus.

Previous work characterizing lotic RNA viromes have found similar results to our survey. The viral orders and families reported in this survey are similar in distribution to RNA viral families reported in urban streams in Quito, Ecuador (Guerrero-Latorre *et al.*, 2018), Singapore (Gu *et al.*, 2018) and central Asia (Alexyuk *et al.*, 2017). This study captured a snapshot of viral communities in the Anacostia River, however we acknowledge that variation in the RNA virome may occur based on any number of physicochemical and meteorological conditions. Van Rossum *et al.* (2018. Spatiotemporal dynamics of river viruses, bacteria and microeukaryotes. *bioRxiv* https://doi.org/10.1101/259861) reported greatest RNA virome temporal variation after rainfall, presumably due to the arrival of runoff from surrounding catchments. This result echoes observations that total virus-like particle abundance experiences greatest variation after rainfall-induced runoff (Williamson *et al.*, 2014) and autecological study of DNA and RNA viruses of soil origin in lake plankton suggested that greatest abundance occurs after sporadic rainfall/storms (Hewson *et al.*, 2012; 2018). Hence, while our findings are consistent with previous work, future study should focus on temporal variation in viromes since it may elucidate potential sources. However, here we discuss three potential sources of viruses based on evidence from this study.

### Anacostia River virioplankton may include fecally-derived viruses

Our survey may support that fecally-derived viruses were present in the Anacostia River at the time of sampling. Evidence of fecally-derived viruses include: recovery of three closely related Dicistrovirus-like genome fragments and four densovirus contigs that were most closely related to viruses retrieved from vertebrate feces (Yao *et al.*, 2021) which may originate from arthropods consumed or from the vertebrate themselves; two nodavirus-like genome fragments that were retrieved in a survey of virioplankton in ports along the Yangtze River estuary (Wolf *et al.*, 2020); a viral contig matching most closely viruses recovered directly from human sewage (Tombusvirus); and a contig in a cluster of viruses that includes those recovered from sewage (Solemovirales). Previous metagenomic study of raw sewage and wastewater effluent recovered all of these groups by viral metagenomics (Ng *et al.*, 2012; Adriaenssens *et al.*, 2018; Pérez-Cataluña *et al.*, 2021), and that these may be concentrated in tissues of filter feeding bivalves and at downstream sites (Adriaenssens *et al.*, 2021). A study by Rothman *et al.* (2021) found both picornaviruses retrieved from invertebrate metatranscriptomes, as well as tobamoviruses in wastewater. Whether these putative fecal viruses remain infective in virioplankton is unknown, however intact plant virions recovered from effluent (Pepper Mosaic and Cucumber Mosaic viruses) were infective in at least one study (Bačnik *et al.*, 2020).

### Anacostia virioplankton bears plant/macrophyte viruses

Next, our survey suggests that plant viruses, including those potentially originating from aquatic macrophytes, may be present in the river virioplankton. Our observation of tombusviruses genome fragments most closely matching viruses of terrestrial plants and a tobamovirus most closely related to the *Chara australis* virus (a freshwater macrophyte) suggest that these may become a substantive part of the Anacostia River virioplankton. Previous work observed a *Chara australis*-like genome fragment in the Finger Lakes, New York suggesting they may be common in freshwater habitats of the northeastern United States (Hewson *et al.*, 2018). Survey by quantitative PCR of this tobamovirus genome revealed that it was below the effective detection threshold of approx. 3 copies mL^–1^ except for one positive detection at site 9 on 27 March 2017 (3×10^4^ copies mL^–1^). These results suggest that while tobamoviruses may constitute a part of virioplankton assemblages, their abundance remains much lower than typical abundances of virus-like particles in freshwater habitats (~10^8^ copies mL^–1^). Our finding of Charavirus detection over time is consistent with Vlok *et al.* (2019) who found that strains of this viral genus can become sporadically abundant in rivers. It is unknown whether *Chara* spp. occurs in the Anacostia River today, but it was recorded in 1925 in the river (McAtee, 1925) and occurs in the downstream Potomac River (Haramis and Carter, 1983).

### Evidence for bivalve viruses in virioplankton

**Fig. 5.**
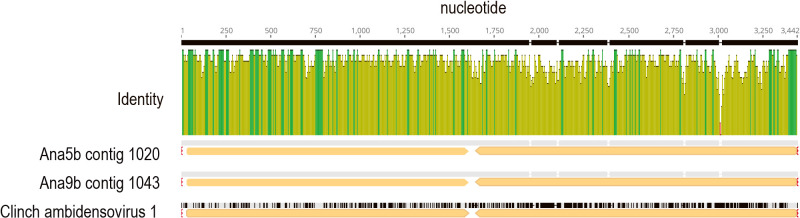
Alignment of Clinch ambidensovirus 1-like fragments recovered from station 5 and 9. The alignment has been trimmed to include the non-structural (left; NS1) and structural (right; VP4) open reading frames. Identity is indicated across 10-nucleotide bins (green=100% identical bins; yellow=<100% identical bins). The mean nucleotide identity was greatest (90.7%) across NS1 and least (83.6%) across VP4. The red bar indicates a 4 amino acid insertion in the VP4 region (Thr-His-Phe-Leu).

Our data also support that mollusks, and potentially freshwater bivalves, may be host organisms for several viral genotypes detected in this survey. At both site 5 and 9, we recovered a densoviral genome fragments sharing >80% nucleotide identity to the capsid protein region of Clinch Densovirus 1 (Richard *et al.*, 2020) (Fig. 5). The similarity of this viral genome fragment to a mussel virus exceeds typical similarity between viruses recovered from different asteroid and arthropod species (Jackson *et al.*, 2020). Additionally, we recovered Picornavirales, Wolframvirales, Solemovirales, and Nodamuvirales genome fragments which clustered with viruses recovered from mollusks. However, these also clustered with phylogenetically disparate hosts. Nevertheless, the recovery of a virus most similar to a mollusk densovirus suggests that bivalves may shed viruses into virioplankton.

Freshwater mussel densities in the Anacostia and nearby Potomac River have declined in the last 5 decades likely due to industrial and urban pollution, mirroring enigmatic declines of freshwater mussels in the eastern United States since the mid-1970s (Wendell, 2019). Introduction of the Asiatic clam *Corbicula fluminea* in the late 1970s to the upper Potomac River led to a shortlived improvement of water quality, but mussel abundances have declined since 1983 (Phelps, 1994). The Clinch densovirus 1 was epidemiologically associated with unionid mussel decline in the Clinch River, Tennessee, which flows westwards from the Appalachian Mountains (Richard *et al.*, 2020). The similarity of a densovirus recovered in this survey to a potentially pathogenic densovirus of mussels raises interesting questions about potential stress on mussel repopulation efforts (Anacostia Watershed Society, 2021).

### Data limitations

Our work is confounded by several potential limitations. First, assigning potential hosts to related viral genome fragments that are not identical may pose challenges to interpretation, since even closely related viruses may infect taxonomically disparate hosts. Hence, while we speculate in this work potential hosts or sources of detected viruses, it is not possible to definitively identify them. Second, viral metagenomic approaches may be beset with reagent and laboratory contaminants which may cloud interpretation (Naccache *et al.*, 2013). Recent metavirome survey of laboratory and reagent contaminants highlighted a number of genome fragments within the viral orders and families reported here (Porter, A.F., *et al.* 2021. Metagenomic identification of viral sequences in laboratory reagents. *bioRxiv* https://doi.org/10.1101/2021.09.10.459871). Because we did not perform blank (reagent) contaminant metaviromes in parallel to these viromes, and since previous blank metaviromes used different reagents, it is possible some of the viral genome fragments reported in this study originate from sources outside our samples. Finally, we capture in this study a snapshot of viromes at three sites and one date, however it is possible that viromes may be subject to short-term (*e.g.* storm) climatic conditions which may influence these results. Hence, overall virioplankton diversity in the Anacostia River may not be adequately explained by this survey alone, and future work is recommended to survey temporal variability in virioplankton structure.

### Conclusions

Despite limitations of our findings, our work suggests that viruses in the impacted Anacostia River virioplankton are influenced by extraneous sources, which may include combined sewage overflows at downstream sites, and that virioplankton at downstream sites may bear signatures of fecally-derived or other terrestrial viruses. Our data also illustrate the potential for Anacostia River virioplankton to comprise viruses originating from invertebrate and plant hosts. Initiatives currently underway to improve water quality and restoration of bivalves in the river should consider monitoring of RNA virioplankton in addition to other chemical and biotic parameters, since virioplankton composition may reflect improved inputs into the lower river, and identify reductions in plant and animal pathogens.

## Citation

Solomon, C., and Hewson, I. (2022) Putative Invertebrate, Plant, and Wastewater Derived ssRNA Viruses in Plankton of the Anthropogenically Impacted Anacostia River, District of Columbia, USA. *Microbes Environ ***37**: ME21070.

https://doi.org/10.1264/jsme2.ME21070

## Supplementary Material

Supplementary Material

## Figures and Tables

**Table 1.** Sites on the Anacostia River and their physical and geographical characteristics (see Solomon *et al.*, 2019 for physicochemical conditions, Velinsky *et al.*, 2011).

Site	Location	Characteristics
Site 1 (Ana1b)	Upper	Close to Bladensburg Water Park; shallow waters with depth <2‍ ‍m with tidal changes
Site 5 (Ana5b)	Middle	Close to the former site of a Pepco power plant; sediments have significant industrial chemical contamination (Tetra Tech, 2019)
Site 9 (Ana9b)	Lower	Close to the most urbanized portion of the river and the confluence of the Anacostia and Potomac rivers; wide and shallow

**Table 2.** Characteristics of metaviromes prepared from the 0.2–0.02‍ ‍m size fraction of plankton in the Anacostia River at sites 1, 5 and 9. RNA viral reads were determined by first comparing contig spectra to an in-house database of RNA and DNA viral proteins collected from NCBI by BLASTx, then verifying their identity by BLASTn against the non-redundant (nr) database. Viral contigs were then subject to read recruitment from each library to determine overall reads associated with viruses. Note that viromes were prepared by amplifying RNA (TransPlex; Sigma-Aldrich), hence quantitative information about relative representation is not provided due to uncertainties in amplification biases between viral groups.

Library	Total Reads	No. Contigs	RNA Viral Reads
Site 1 (Ana1b)	4,483,108	1,225	960,892
Site 5 (Ana5b)	3,686,906	7,428	755,972
Site 9 (Ana9b)	6,145,162	5,568	1,491,700
