# Supplementary material for: Putative Invertebrate, Plant, and Wastewater Derived ssRNA Viruses in Plankton of the Anthropogenically Impacted Anacostia River, District of Columbia, USA
Source: Microbes Environ. 2022 Mar 10;37(5):ME21070. doi: 10.1264/jsme2.ME21070 (PMC9763036; doi:10.1264/jsme2.ME21070)
Supplement: Supplementary file 1 — Supplementary Material [file 37_21070_s1.pdf]

**Supplemental Table 1:** Primer, probe and oligonucleotide sequences used to quantify two RNA viral genotypes using quantitative reverse-transcriptase PCR (qRT-PCR).

| **Target** | **Oligonucleotide** | **Sequence (5' – 3')** |
|---|---|---|
| Stn9 contig 1041 | Forward | TGAAGCTGACGGACTACGTG |
| | Reverse | CGGCACTGTGCAAGAGATAA |
| | Probe | TCCACACGGGCATTGTGAATGAGT |
| | Standard | ATGAAGCTGACGGACTACGTGTCCACACGGGCATT GTGAATGAGTTTATCTCTTGCACAGTGCCGG |
| Stn9 contig 1251 | Forward | CCTCTGCATCCAGACACTGA |
| | Reverse | GGTGCTTGACACACACACAACC |
| | Probe | GACGTCTTCAGGTCTCGTTGTGGGT |
| | Standard | ACCTCTGCATCCAGACACTGACGACGTCTTCAGGT CTCGTTGTGGGTTTGTCTACGTATGTGGTTGTGTGT GTCAAGCACCA |

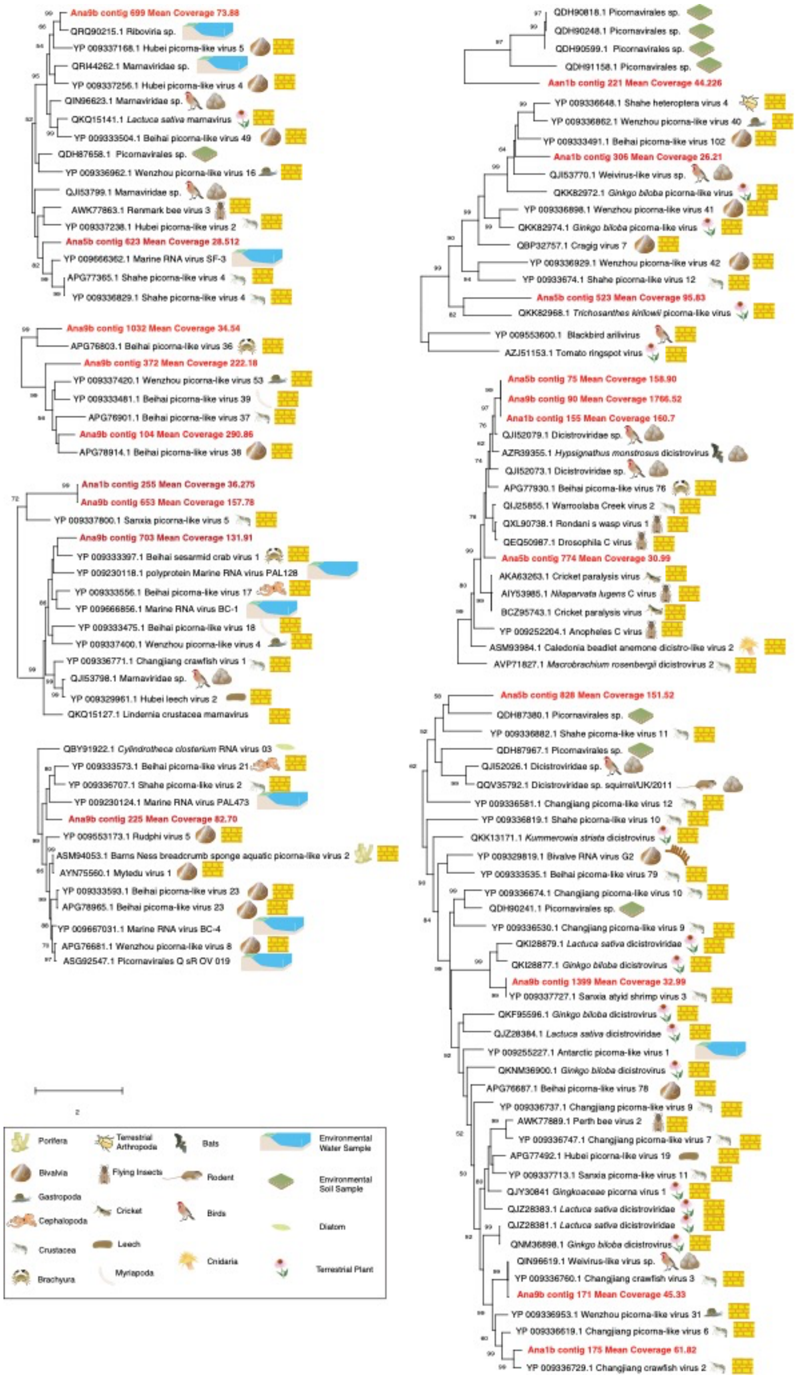


**Supplemental Fig. 1**: Phylogenetic analyses of Picornavirales-like genome fragments (>2000nt and at >20X coverage) recovered from the Anacostia River. The analysis was based on a 430 amino acid alignment, using the general reverse transcriptase model of substitution (Dimmic et al., 2002) and gamma distribution of rates among sites where each tree includes the closest sequences to the Anacostia sequences at NCBI. The putative source/host and sample type of each matching sequence is provided with symbols. Collapsed nodes indicate branches which did not contain sequences similar to contigs recovered from the Anacostia River.

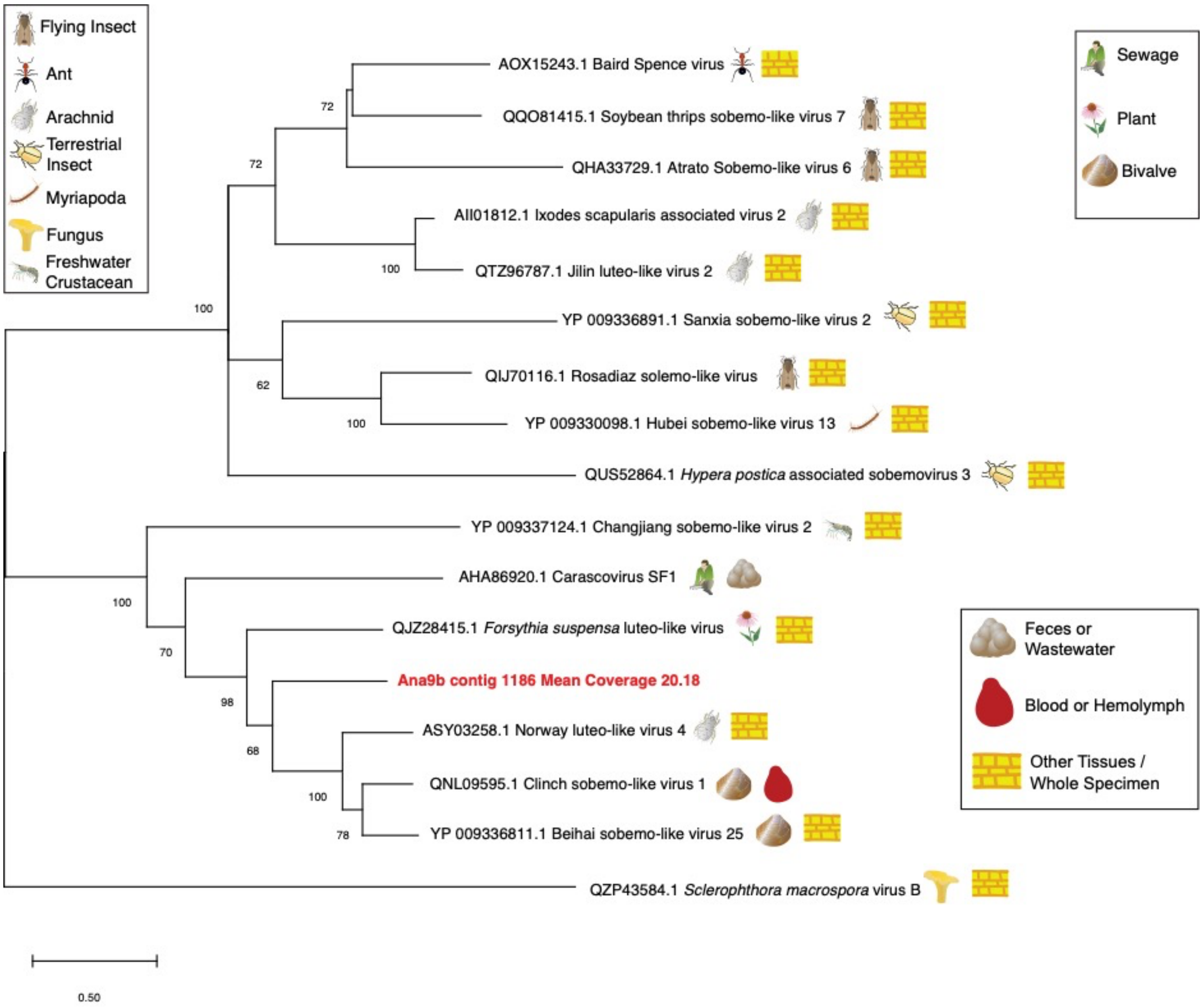


**Supplemental Figure 2:** Phylogenetic analyses of Solemivirales-like genome fragments (>1500nt and at >15X coverage) recovered from the Anacostia River, based on a 241 amino acid alignment, using the LG model (Le and Gascuel, 2008) and gamma distribution of rates among sites which includes the closest sequences to the Anacostia sequences at NCBI.  The putative source/host and sample type of each matching sequence is provided with symbols.

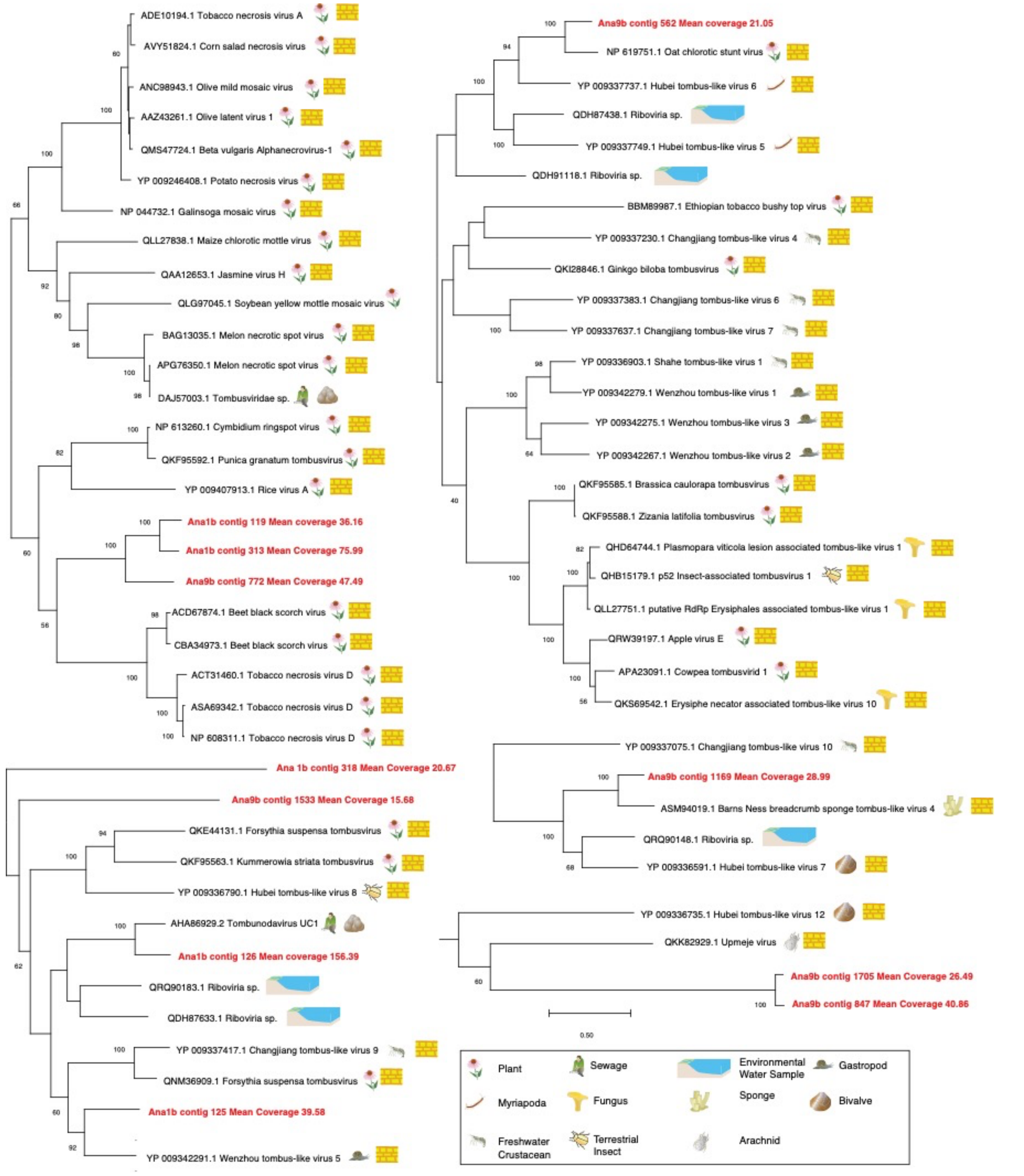


**Supplemental Figure 3:** Phylogenetic analyses of Tolivirus-like genome fragments (>1500nt and at >15X coverage) recovered from the Anacostia River. The analysis was based on a 244 amino acid alignment, using the general reverse transcriptase model of substitution (Dimmic et al., 2002) and gamma distribution of rates among sites where each tree includes the closest sequences to the Anacostia sequences at NCBI. The putative source/host and sample type of each matching sequence is provided with symbols.

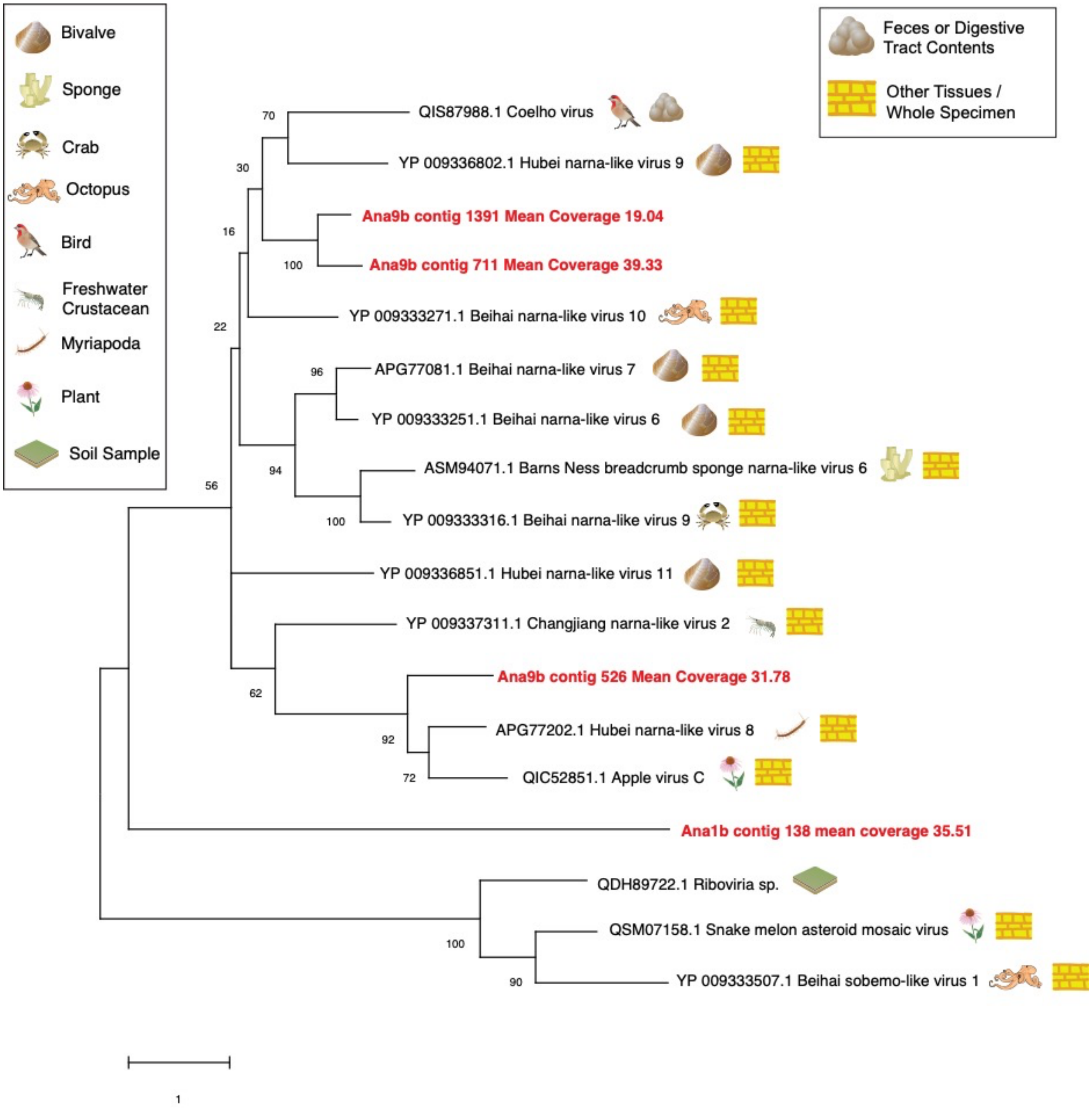


**Supplemental Figure 4:** Phylogenetic analyses of Wolframvirales-like genome fragments (>1500nt and at >15X coverage) recovered from the Anacostia River, based on a 274 amino acid alignment, using the general reverse transcriptase model of substitution (Dimmic et al., 2002) and gamma distribution of rates among sites and including the closest sequences to the Anacostia sequences at NCBI. The putative source/host and sample type of each matching sequence is provided with symbols.

**REFERENCES**

Dimmic MW, Rest JS, Mindell DP, Goldstein RA (2002) rtREV: an amino acid substitution matrix for inference of retrovirus and reverse transcriptase phylogeny. J Molec Evol **55:** 65-73

Le SQ, Gascuel O (2008) An improved general amino acid replacement matrix. Molec Biol Evol **25**: 1307 - 1320
